# Pandemics, pathogenicity and changing molecular epidemiology of cholera in the era of global warming

**DOI:** 10.1186/s12941-017-0185-1

**Published:** 2017-03-07

**Authors:** Fazle Rabbi Chowdhury, Zannatun Nur, Nazia Hassan, Lorenz von Seidlein, Susanna Dunachie

**Affiliations:** 1 Peter Medawar Building for Pathogen Research, University of Oxford, South Parks Road, Oxford, OX1 3SY UK; 2 Centre for Tropical Medicine and Global Health, University of Oxford, Oxford, UK; 3 Health Directorate, Dhaka, Bangladesh; 4 Yarrawonga Health and Yarrawonga Medical Centre, Yarrawonga, Victoria 3730 Australia; 5 Department of Pathology, Dhaka Community Medical College, Dhaka, Bangladesh; 6 Mahidol-Oxford Tropical Medicine Research Unit, Bangkok, Thailand

**Keywords:** Cholera, Climate change, Epidemiology, Global warming

## Abstract

**Background:**

*Vibrio cholerae,* a Gram-negative, non-spore forming curved rod is found in diverse aquatic ecosystems around the planet. It is classified according to its major surface antigen into around 206 serogroups, of which O1 and O139 cause epidemic cholera. A recent spatial modelling technique estimated that around 2.86 million cholera cases occur globally every year, and of them approximately 95,000 die. About 1.3 billion people are currently at risk of infection from cholera. Meta-analysis and mathematical modelling have demonstrated that due to global warming the burden of vector-borne diseases like malaria, leishmaniasis, meningococcal meningitis, viral encephalitis, dengue and chikungunya will increase in the coming years in the tropics and beyond.

**Cholera and climate:**

This review offers an overview of the interplay between global warming and the pathogenicity and epidemiology of *V. cholerae*. Several distinctive features of cholera survival (optimal thriving at 15% salinity, 30 °C water temperature, and pH 8.5) indicate a possible role of climate change in triggering the epidemic process. Genetic exchange (ctxAB, zot, ace, cep, and orfU) between strains and transduction process allows potential emergence of new toxigenic clones. These processes are probably controlled by precise environmental signals such as optimum temperature, sunlight and osmotic conditions. Environmental influences on phytoplankton growth and chitin remineralization will be discussed alongside the interplay of poor sanitary conditions, overcrowding, improper sewage disposal and global warming in promoting the growth and transmission of this deadly disease.

**Conclusion:**

The development of an effective early warning system based on climate data could help to prevent and control future outbreaks. It may become possible to integrate real-time monitoring of oceanic regions, climate variability and epidemiological and demographic population dynamics to predict cholera outbreaks and support the design of cost-effective public health strategies.

## Background

*Vibrio cholera* (*V. cholerae*), which causes outbreaks resulting in massive disease burden, is a Gram-negative, non-spore-forming curved rod. *V. cholerae* is an autochthonous member of diverse aquatic ecosystems around the planet, and is often found in close association with a variety of algae and crustaceans [1]. The World Health Organization estimates that officially reported cases of cholera represent only 5–10% of the actual case number occurring annually worldwide [2]. A recent spatial modelling technique estimated that around 2.86 million cholera cases occur globally every year, and of them approximately 95,000 die [3]. About 1.3 billion people are currently at risk of being infected with cholera [3].

**Fig. 1:**
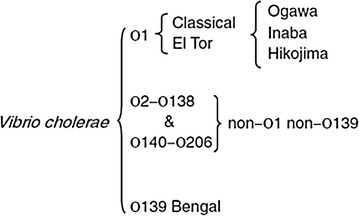
Classification of *Vibrio cholera*. *V. cholerae* is classified firstly by serotype. O1 is subdivided into two biotypes, classic and El Tor on the basis of phenotypic characteristics (although genetic hybrids between the *V*. *cholerae* O1 classic and El Tor biotypes have been reported). Organisms in both biotypes are further classified according to the presence of somatic antigens into two major serotypes (Inaba and Ogawa) and an unstable intermediate type (Hikojima)

The bacterium is classified by the composition of its major surface antigen (O) from lipopolysaccharide into serogroups, of which there are nearly 206 [4]. Only two serogroups of *V. cholerae*, O1 and O139, have been considered causative agents of cholera [4]. The *V. cholerae* O1 strain is further divided into two biotypes named El Tor and classical, on the basis of biochemical differences and bacteriophage susceptibility (Fig. 1). It is generally accepted that seven distinct cholera pandemics have occurred since the onset of the first report in 1817 and the seventh one in 1961 in Egypt [5]. It reappeared dramatically and unexpectedly in January 1991 in Latin America as a continuation of seventh pandemic after a hiatus in that region of more than a century [6]. The appearance in late 1992 in southern India of an epidemic strain of *V. cholerae*, O139 Bengal has caused global concern [7, 8]. This concern was probably unwarranted as the prevalence of O139 cases seems to be receding. It has been hypothesised that the serological switching and genetic diversity of strains between pandemics and epidemics may be influenced by climate variations, with global warming making the environment favourable for cholera outbreaks by switching of virulence factors. This hypothesis is supported by studies of *V. cholerae* outbreaks in India [9], Bangladesh [10] and China [11], as well as a literature review of the influence of environmental factors on *V. cholerae* presence [12]. Furthermore, experimental studies have demonstrated variation with changes in environmental conditions in expression of ChiA2 and TfoX, key regulators of *V. cholerae* horizontal gene transfer [13]. Warmer conditions also provide enriched food sources and ecological protection for the organism by causing algae blooms and making availability of chitin. The delta region of the Ganges and the Brahmaputra have been identified as cholera’s ‘native habitat’ for centuries, and the source for periodic pandemic spread [14]. Of the 36 countries that reported cholera in the seventh pandemic, 28 were newly affected countries and 16 were in Africa [15]. Outbreaks or cases have since been reported in Pakistan, Nepal, China, Thailand, Kazakhstan, Afghanistan, and Malaysia, Sierra Leone, The Democratic Republic of Congo, Nigeria, Angola and Zimbabwe [16–20]. Since the beginning of the century the cholera prevalence has massively expanded on the African continent. In 2015 the majority of cholera cases and mortality were reported from sub-Saharan Africa [21]. Imported cases have been reported in the United Kingdom and United States amongst other countries and in Haiti after the massive earthquake [17, 22–24].

The Haiti outbreak in 2010 was thought to be imported from South Asia by United Nations peace keeping forces [24–26]. Analysis of local climatic variables suggested that following importation, other factors contributed to propagation of the outbreak. The average air temperature and rainfall preceding the outbreak in Haiti was significantly above the long term average of that region [27]. In addition, destruction of sanitary infrastructure and lack of safe water made the situation more vulnerable for the habitant [27]. The cholera outbreak in Haiti supports the suggested role of climatic factors in the transmission of cholera. This review will explore the interplay between environmental influences, changing virulence factors and the molecular epidemiology of cholera.

## Variation of strain (serotype and biotype) during epidemic and pandemic

The *V. cholerae* strain type causing the first four pandemics are unknown. The fifth and sixth pandemics were caused by the classical biotype of O1 strains whereas the seventh was due to the El Tor biotype. In 1992 an epidemic clone of a non-O1 strain with serogroup O139 Bengal caused a large cholera outbreak in Bangladesh and neighbouring countries [8, 28]. At the beginning of the outbreak, the O139 strain displaced the *V. cholerae* O1 strains, including both classical and El Tor biotypes, which coexisted only in Bangladesh. The subsequent emergence of a new clone of *V. cholerae* O1 El Tor that transiently displaced the O139 strains during 1994 and 1995, and the re-emergence in 1996 of *V. cholerae* O139 as the main cause of cholera in Calcutta and its coexistence with the O1 El Tor strains demonstrated temporal changes in the epidemiology of cholera [29, 30]. These clones are genetically (different ctx genotype and ribotype) different compared to other co-existing global clones (the Mexican Gulf coast clone, and the Australian clone) [31].

If we consider these events from a different angle, this heterogeneity of clones might not be due to serological switching but emergence of a new toxigenic strain. Transient appearance and disappearance of more than six ribotypes of classical *Vibrios* [32–34], at least five ribotypes of El Tor *Vibrios* [31], and three different ribotypes of *V. cholerae* O139 [35, 36] during epidemics between 1961 and 1996 in Bangladesh have been demonstrated. Genetic diversity among clinical and environmental isolates during and between epidemics over the past 20 years were also found [34, 37]. All these studies indicate that there has been a continual emergence of new clones of toxigenic *V. cholerae* replacing existing clones. The question is what factors contribute to this shifting? We hypothesize that the clonal shifting in *V. cholerae* is driven chiefly by environmental variations rather than by human-borne dissemination.

## Virulence factor and transduction

Naturally occurring strains of toxigenic *V. cholerae* O1 and O139 contain the virulent enterotoxin cholera toxin (CT). The genes encoding CT (ctxAB) are part of a larger genetic element (CTX genetic element) consisting of at least six genes (ctxAB, zot, ace, cep, and orfU) [38]. The entire CTX element constituted the genome of a filamentous bacteriophage (CTXф) [38]. Genetic exchange in the environment allows the potential emergence of new toxigenic clones by lysogenic (self-replicating element) conversion with a filamentous bacteriophage (CTXф). CTXф encodes cholera toxin including its receptor, and the toxin-co regulated Pilus (TCP), another important virulence factor [38]. CTXф confers increased evolutionary fitness to its host and hence to its own nucleic acids. With growing immunity in the host population against certain toxigenic clones of *V. cholera*, new toxigenic clones emerge and replace existing clones by a process of natural selection. Novel clone emergence may also happen by transduction. Transduction is the process by which DNA is transferred from one bacterium to another by a bacteriophage. Genes for both TCP and CTX (virulence factor) can be readily transduced into recipient strains via temperate phages [29]. Induction of CTXф lysogens is probably controlled by precise environmental signals such as optimum temperature, sunlight, and osmotic conditions [29]. Exposure to sunlight is a key factor in the induction of CTX prophase from the host and subsequent propagation of the phage particle [27, 29, 39]. The continual emergence of new strains of toxigenic *V. cholera* and their selective enrichment during cholera outbreaks constitute an essential component of the ecosystem for their survival. Goel et al. investigated two subsequent epidemics in India by genomic finger printing analysis. They have identified heavy rainfall as the plausible cause of genotype switching between O1 and O139 type strain. The duration between two peaks and switching was only 2 weeks [9]. Koelle et al. also showed in Bangladesh through a phenotypical modelling that shifting in *V. cholerae* serotype is driven chiefly by environmental variations rather than by human-borne dissemination [10]. The reason behind is the pathogen’s sensitivity to environmental fluctuations. El Tor, a seasonal generalist, seems to gain an advantage over the classical strain, a seasonal specialist, when monsoon rainfall becomes more extreme [10]. This is because El Tor is known to be more fit then classical type because of the expression of vibrio polysaccharide (vps) gene which provide a protection to them against climatic variables [10]. This molecular difference therefore establishes the trade-off between maximum transmission and sensitivity to climatic fluctuations between the serotypes [10].

## Presence of virulence factors in environmental strain

Another alarming feature for cholera control is the presence of these virulence factors in environmental or non-toxigenic strains (non O1 and non-O139) of *V. cholerae*. Ghosha et al. identified the presence of virulence-associated genes like ctxA, tcpA, toxR and the repetitive sequence (RS element) in both non O1 and non-O139 strains [40]. They suggested that a new toxigenic strain could evolve from these strains in favourable conditions. Kirschner et al. also observed rapid growth of non O1 and non-O139 strain in the presence of dissolved organic carbon and water temperature [41]. It is therefore a major challenge to determine the role of these seemingly innocuous inhabitants in the development of new pathogenic strains capable of causing outbreaks.

## Favourable environmental conditions

*Vibrio cholerae* thrive in 15% salinity, 30 °C water temperature, and pH 8.5 [42]. In the absence of these optimal conditions it is difficult to isolate toxigenic strains from previously endemic regions. By contrast, epidemic periods occur during warmer water temperatures in combination with elevated pH and plankton blooms, and it becomes easy to isolate toxigenic strains [42]. Montilla et al. found conversion from non 01 to 01 and vice versa in all microcosms of both artificial sea water and natural water at various temperatures [42]. They concluded that seroconversion from non-O1 to O1 occurred earliest (within 5 days) at a salinity of ~10% and temperatures near 35 °C [42]. A study in Bangladesh revealed that the highest number of clinical cases occur when the air temperature is more than 28.66 °C and sunshine is more than 4.13 h/day [43]. Increased environmental water temperatures decrease multiplication time and hastened growth along with an increased detection rate of *V. cholera* [29]. This suggested a triggering factor which may be responsible for enhancing the number of organisms under certain environmental conditions [29]. *V. cholerae* O1 El Tor and O139 are able to form a three-dimensional biofilm on surfaces, providing a microenvironment [12]. Within the biofilm, the organisms can survive during inter-epidemic periods. Biofilm formation is dependent on the expression of an exopolysaccharide (EPS). Expression of the vps synthesis genes, encoded in two gene clusters on the larger chromosome (vpsA-K and vpsL-Q), is required for the synthesis of EPS [12]. These gene clusters show higher expression at the time of increased salinity and water temperature [12]. Lu et al. in China also found that, spatiotemporal serotype shifts (Ogawa, Inaba and O139) generally correlated with the variations in the pulsed-field gel electrophoresis patterns (PIV, PIIIc, PIa, PIIIb, PIIIa, PIb, and PII) [11]. These patterns largely influenced by seasonal variations particularly rainfall due to formation of bio film [11].

## Plankton load and role of chitin

Sunlight, temperature, and nutrients all influence the growth of phytoplankton (green, red and blue-green) and aquatic plants, which in turn raise the pH of the surrounding water to favour growth of *V. cholera* [44]. Blue-green algae are a reservoir of *V. cholera* [44]. Heavy loads of phytoplankton produce food for zooplankton in the next level of the food chain. Various chitinous organisms (whose exoskeleton is formed by the polysaccharide chitin), e.g. copepods, amphipods, and other crustaceans (crabs, lobsters and shrimp) are prevalent among zooplankton populations [45]. Chitin is thin and provides flexibility to these organisms [45]. If chitin is not degraded, high levels of carbon and nitrogen remain insoluble and inaccessible to most organisms [29]. Using chitinase, marine bacteria including *Vibrio* spp. play a significant role in chitin remineralisation, so very little chitin can be detected in aquatic sediments [29]. In addition to providing a food source for *Vibrios* and enhancing survival under starvation conditions, chitin also offers protection to *V. cholerae* at low temperatures and under acidic conditions such as the human gut. This could be important if copepods carrying *V. cholerae* are ingested in drinking water [29]. Such protection is likely to play an important role in the expanding and changing epidemiology of cholera.

Recent studies suggest that chitin facilitates and regulates competence-mediated horizontal gene transfer in *V. cholera* by expressing Chi A2 and TfoX [13, 46]. The seroconversion between O1 El Tor and O139 Bengal during the 1992 epidemic in Bangladesh and India could be due to this factor [46]. The activity of Chi A2 and the expression of TfoX gene could be influenced by environmental conditions such as high salinity and water temperature [13].

## Other environmental factors

Other influences under investigation include sea surface height, river discharge, climate variables that affect water levels and salinity [47]. Cholera is primarily a waterborne disease. Sanitation facilities, provision of safe drinking water and good infrastructure for sewage treatment in industrialized nations has made cholera extremely rare. Outbreak studies done in Odisha, India found a clear relationship between sewage contaminated water and cholera epidemics [48]. Several field surveys in West Bengal and Gujrat revealed cholera outbreaks related to sewage leaks from municipality pipelines and local overpopulation [49–51]. In Africa, water source contamination due to weak sanitary infrastructure is considered the main risk factor for cholera [52]. It is inevitable that poor sanitary conditions, overcrowding with the majority of people living below the poverty line, lack of proper sewage disposal systems and improper water supply, all provide ideal transmission conditions for cholera once it is introduced into a community. In future as an effect of global warming these conditions could worsen in many parts of the world.

## Prevention by vaccine

An extensive review of vaccines is beyond the scope of this review, but the benefits of vaccinations merit highlighting. Although the ideal vaccine is yet to be developed, some promising vaccines have been licensed. Among them oral killed cholera vaccines (OCVs) are particularly helpful in the control and prevention of outbreaks. A systematic meta-analysis found the overall vaccine efficacy was 52% during the first year and 62% during the second year with less protectiveness in children under 5 years of age [53]. Other limitations to this approach are the necessity of multiple doses, limited evidence of long term protection, production costs etc. [54]. Single dose administration of oral vaccine is also protective and can reduce the logistic requirements for mass vaccination campaigns [55]. A single dose of the inactivated/killed oral vaccine, ‘Shanchol’ was found to be efficacious in older children (≥5 years of age) and in adults in Bangladesh which is a high cholera endemic setting [55]. Though the currently available and licensed cholera vaccines do not confer life-long 100% protection they are increasingly recognised as valuable tools in the containment of cholera outbreaks. New funding mechanisms and the creation of the international cholera vaccine stockpile are helping to make OCVs globally available [56].

Recent research has focused on alternative delivery systems such as micro particles, proteoliposomes, LPS subunit, DNA vaccine and rice seeds containing toxin subunits [57]. Although a fully ideal vaccine has yet to be designed, these new approaches show promise.

## Future directions

Analysis of global temperature data led the Inter-Governmental Panel for Climate Change to the conclusion that the average global temperature over land and ocean surfaces has risen by 0.85 °C (0.65–1.06) in the period from 1880 to 2012 [58]. They predicted that global surface temperature will rise by 1.5 °C relative to 1850 by the end of the twenty-first century (2081–2100) [58]. Climatologists also estimate that the sea level has risen by about 1 mm/year in recent decades, caused by thermal expansion [29]. These events will change the diversity of cholera epidemiology throughout the world in the future. Spontaneous seroconversion and rapid transduction between environmental and toxigenic strains giving rise to the emergence of new epidemic strains could become a global issue. How this happens, and what the impact of climate change will be is a matter of ongoing debate. The potential risk of the presence of virulence factors in different environmental (non-toxigenic) strains as well as the possible role of global warming is an important area for future research.

## Conclusion

Cholera cannot be imminently eradicated as it is a native species of the aquatic eco system. It is found in close association with various algae and crustaceans which are also natural inhabitants of water. But the disease is preventable with implementation of public health measures to ensure adequate sanitation and safe water supply. Climate change is challenging the health sector. Meta-analysis and mathematical model have demonstrated that the burden of vector borne diseases like malaria, leishmaniasis, meningococcal meningitis, viral encephalitis, dengue, chikungunya, giardiasis, toxoplasmosis etc. will increase in coming years in the tropics and beyond [59, 60]. The licensing of several oral cholera vaccines has added a promising tool to the traditional cholera control strategies namely safe water, sanitation and hygiene improvements.

With changing climate patterns more countries may become at risk of cholera, and there is a high risk of further pandemics. Interdisciplinary analysis and integrated prevention strategies are mandatory. Mathematical modelling of the disease in relation to climate offers a first step to identify the key environmental and climate parameters associated with disease variability. The development of an effective early warning system based on climate data could help to prevent and control future outbreaks. It may become possible to integrate real-time monitoring of oceanic regions, climate variability and epidemiological and demographic population dynamics to predict cholera outbreaks and support the design of cost-effective public health strategies.

## Abbreviations

*V*. *cholerae*: *Vibrio cholerae*
CT: cholera toxin
TCP: toxin co-regulated pilus
RS: repetitive sequence
OCVs: oral cholera vaccines
EPS: exo poly saccharide
